# Human-centered AI in healthcare teams: integrating clinical oversight, EHR auditability, and reimbursement pathways for responsible adoption

**DOI:** 10.3389/fdgth.2026.1761009

**Published:** 2026-07-21

**Authors:** Michael C. Changaris, Franca V. Niameh

**Affiliations:** 1 Integrated Health Psychology Program, The Wright Institute, Berkeley, CA, United States; 2 Contra Costa Family Medicine Residency Program, Contra Costa Health, Martinez, CA, United States; 3 Family Medicine Residency Program, John Muir Health, Walnut Creek, CA, United States

**Keywords:** artificial intelligence, value-based care, integrated behavioral health, electronic health records, clinical decision support, health policy, reimbursement models, implementation science

## Abstract

**Background:**

AI is being introduced into clinical workforces during a critical transition toward integrated, value-based models of care, where its greatest promise lies in augmenting clinician judgment and expanding the reach of already strained healthcare teams. Yet clinical adoption remains limited because most AI systems lack reimbursement pathways, impose substantial implementation costs, and lack standardized mechanisms for integration into electronic health records (EHRs). These gaps create misalignment between technological capability and clinical usability. This paper identifies financial, regulatory, and workflow structures required for AI to operate safely, predictably, and sustainably across key domains of healthcare.

**Methods:**

This narrative synthesis reviews clinical, economic, regulatory, and implementation-science literature from 2022 to 2025. Four domains were analyzed: (1) AI augmentation of clinical workflows; (2) reimbursement structures and CPT coding pathways; (3) EHR-based AI deployment and governance; and (4) economic and equity considerations for large-scale implementation. Sources included peer-reviewed reviews, white papers, consensus statements, and health policy analyses.

**Results:**

AI tools demonstrated benefits in diagnostic accuracy, decision support, and documentation efficiency, particularly in radiology, cardiology, and EHR-integrated workflows. Adoption was hindered by absent reimbursement for clinician-reviewed AI outputs. Implementation and monitoring costs fell heavily on health systems, risking widened disparities. Additional concerns included accuracy, bias, generalizability, and limited oversight. Enabling conditions included clinician-in-the-loop review, auditable outputs, equity-centered validation, and alignment with evolving payment models.

**Conclusions:**

AI can strengthen healthcare teams and advance value-based care, but scaling requires aligned incentives, rigorous evaluation standards, equity safeguards, and coordinated governance—echoing lessons from national EHR implementation.

## 1 Introduction

Artificial intelligence (AI) is entering clinical workforces at a pivotal moment in team-based care and the transition to value-based care, bringing transformative potential to enhance and augment care teams, not by replacing clinicians, but by amplifying their expertise and extending their reach. AI already shows promise in improving diagnostic accuracy, strengthening care coordination, and increasing documentation efficiency. Yet without clear reimbursement pathways, governance standards, and safeguards for equity and safety, AI risks widening existing disparities and creating opaque decision processes that undermine clinician trust. Realizing AI's benefits will depend on implementing tools transparently, auditing them rigorously, and ensuring they align with value-based care rather than functioning as unregulated add-ons to already burdened workflows.

Adoption, however, remains uneven because the U.S. healthcare system still relies heavily on fee-for-service reimbursement and lacks clear mechanisms to evaluate, compensate, or govern AI-enabled clinical work. As payment shifts toward value-based care (VBC), AI could strengthen team-based, outcomes-oriented practice, but only if supporting structures evolve. Emerging evidence suggests that scalable implementation depends on four interdependent elements: (1) reimbursement pathways that recognize clinician-reviewed AI outputs, (2) EHR integration that supports auditability and workflow fit, (3) governance standards that promote accuracy, safety, and equity, and (4) ongoing performance monitoring to assess generalizability and detect bias (83). This paper examines how these elements shape AI's role within VBC and the policy conditions needed for safe and equitable adoption.

The broader context for AI adoption is the restructuring of U.S. healthcare financing. Fee-for-service (FFS) historically rewarded service volume, fragmented care, and overlooked behavioral and social drivers of health (1). AI is increasingly viewed not as a replacement for clinicians but as a computational extension of team-based care.

Prior AI policy and ethics reviews have largely focused on algorithmic performance, regulation, and high-level governance, often treating reimbursement, workflow integration, and clinician accountability as secondary implementation issues. This review advances the field by positioning reimbursement alignment, EHR auditability, and clinician supervision as foundational requirements for responsible clinical AI. We argue that without auditable, clinician-reviewed workflows that fit existing payment models, AI systems are unlikely to scale safely or equitably. Using Integrated Behavioral Health (IBH) and Primary Care Behavioral Health (PCBH) as implementation testbeds, this paper grounds policy analysis in care models where team-based workflows, measurement, and value-based reimbursement are already established.

Federal policy has accelerated this transition. The Medicare Access and CHIP Reauthorization Act of 2015 (MACRA) restructured Medicare physician reimbursement through the Merit-Based Incentive Payment System (MIPS) and Advanced Alternative Payment Models (APMs), linking payment to quality, digital infrastructure, population health, and cost control. By 2018, CMS projected that most Medicare FFS payments would be tied to quality and that half would flow through APMs, establishing VBC as the prevailing trajectory for U.S. healthcare.

Team-based care is central to this transformation. Collaborative practice across physicians, nurses, behavioral health clinicians, pharmacists, social workers, and care managers enables organizations to address multidimensional patient needs. Team-based care, defined as two or more health professionals working jointly with patients and caregivers toward shared goals, advances the Triple Aim of improving experience, population health, and cost efficiency (2, 3). Evidence suggests that well-functioning teams enhance chronic disease management, reduce hospitalizations, and strengthen longitudinal relationships foundational to whole-person care (4).

Successful implementation would benefit from healthcare organizations building capacity in three interconnected domains: organizational capabilities, provider engagement (81), and patient–family–community engagement. For academic medical centers, these efforts could be woven into educational and research missions, while maintaining tertiary care responsibilities across broad geographic regions. Organizational redesign efficacy will be enhanced if it supports all clinicians in practicing at the top of their license while investing in workflow improvements, quality infrastructure, and sustained change management (5).

Artificial intelligence (AI) is increasingly described as a possible extension of team-based care within value-based systems. Existing studies suggest that AI typically augments, rather than replaces, clinicians by highlighting potential risk patterns, helping extract information from narrative text, and offering decision-support that may contribute to diagnostic safety. When integrated into electronic health records (EHRs), AI tools have been used to flag hospitalization risk, identify preventive care gaps, detect behavioral health needs not captured through structured screeners, and surface social determinants of health relevant to care planning (6, 95). In specialty and accountable care settings, NLP and predictive models have been associated with more proactive outreach, adjustments in treatment planning, and reductions in avoidable emergency department use (7–9).

AI may also provide support in areas that can be challenging for value-based care, such as documentation and coordination. Ambient documentation tools have been linked to reductions in clerical burden and improved structured data capture (10, 89). AI-enabled referral management systems can help identify missed specialty or behavioral health follow-ups, potentially supporting continuity in integrated behavioral health (IBH) settings, and exploratory work in academic detailing suggests a role for AI in surfacing guideline-concordant recommendations and prescribing trends (11, 12).

Despite recent progress, most AI tools remain outside reimbursement pathways. Their outputs, including risk alerts or SDOH identification, generally do not match existing CPT or HCPCS codes (13). As a result, implementation tends to be more feasible in well-resourced systems, while community health centers, rural clinics, and safety-net providers may face barriers to adoption. This uneven access raises concerns about potential widening of the digital divide and its implications for equity within value-based care (14).

**Figure 1:**
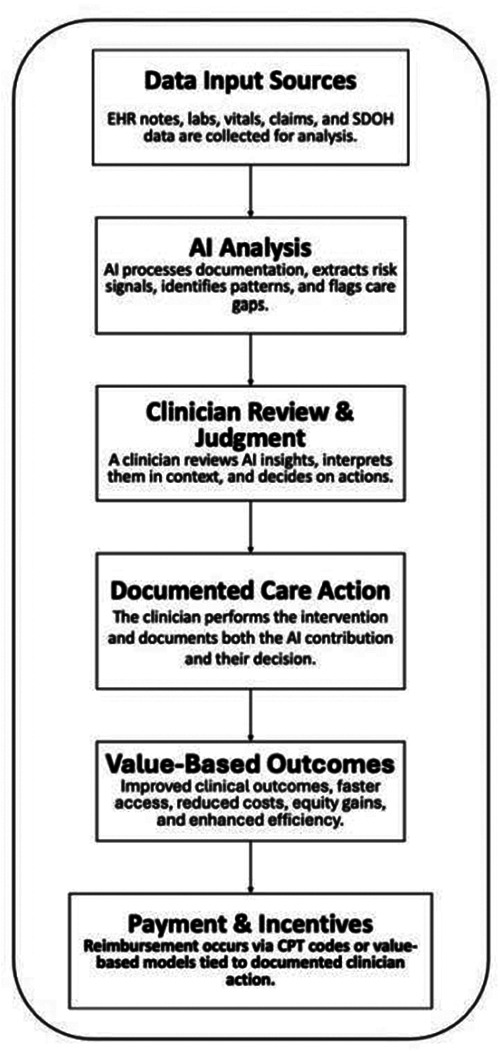
AI-Supported clinical decision and reimbursement pathway. Illustrates the operational workflow required for reimbursement: AI generates non-billable computational output, a clinician performs the interpretive and accountable action, and documented care then links to value-based outcomes and payment. This framing distinguishes machine work from clinician work in a way payers can operationalize.

As illustrated in Figure 1, AI-Supported Clinical Decision and Reimbursement Pathway. AI contributes computational insights that require clinician review, and the clinician's documented action creates the auditable link to outcomes and reimbursement.

Early evidence suggests that primary care may be especially well positioned to benefit when AI strengthens relationship-centered care, enhances early detection, and supports teams caring for complex populations (15). Large-scale AI deployment is unlikely to meaningfully improve care if introduced into workflows that are already misaligned. Fragmented EHR systems, inconsistent financial incentives, communication barriers, and limited patient-engagement models all restrict what AI can realistically achieve.

The transition to AI-enabled value-based care may also require cultural and regulatory shifts. Medical education may need stronger alignment with system-level goals such as population health and quality improvement (16). Organizations can prepare teams to use AI in ways that reinforce, rather than compete with, patient–clinician relationships. Governance structures addressing accuracy, bias, explainability, and liability may further support trust and safe implementation (83).

The movement from volume-based care to valuing outcomes offers an important opportunity (1, 17), but without reimbursement pathways that recognize clinician-reviewed AI outputs, benefits will likely remain uneven, exacerbating existing inequities (96). Embedding AI outputs within billable, auditable workflows may be key to ensuring these tools advance, rather than undermine, the goals of value-based care.

This review proceeds in three stages. First, we situate clinical AI within the policy and delivery context of value-based, team-based care, with particular attention to reimbursement structures, governance, and equity considerations. Second, we introduce three complementary frameworks: (1) a billing and policy framework for reimbursable, auditable AI in value-based integrated care; (2) a workflow model illustrating AI-augmented Primary Care Behavioral Health (PCBH); and (3) a policy and implementation roadmap outlining requirements for governance, equity assurance, and workforce readiness. Together, these frameworks provide a structured approach for translating responsible AI principles into operational, reimbursable clinical practice.

## 2 Methods policy review

### 2.1 Approach

This manuscript employs an integrative policy synthesis approach (116) to develop a reimbursement framework for AI-augmented care delivered through electronic health record (EHR) systems. The objective was to integrate evidence from health informatics, reimbursement policy, value-based care, regulatory guidance, and implementation science to identify pathways for recognizing AI-mediated clinical activities within existing billing structures (91).

Given the rapid evolution of digital health reimbursement and the cross-disciplinary nature of AI governance, this methodology uses purposive, concept-driven source selection to support coherent conceptual integration across heterogeneous evidence types. It synthesizes empirical trials, consensus frameworks, regulatory guidance, and theoretical policy analyses to identify actionable pathways within existing U.S. healthcare billing structures. The objective is structured conceptual integration rather than quantitative aggregation.

### 2.2 Evidence gathering and search strategy

A targeted review of peer-reviewed literature, federal and state policy documents, and regulatory guidance published between 2015 and 2026 was conducted. Searches were performed in PubMed, Scopus, Web of Science, Google Scholar, and policy repositories including the Centers for Medicare and Medicaid Services (CMS), Agency for Healthcare Research and Quality (AHRQ), National Committee for Quality Assurance (NCQA), and state Medicaid waiver archives. Searches combined terms covering four analytic domains:

- **AI in clinical workflows:** “clinical decision support,” “predictive analytics,” “natural language processing,” “risk stratification,” “EHR-embedded AI.”
- **Reimbursement and economics:** “AI reimbursement,” “digital health payment models,” “CPT codes,” “care-management billing,” “value-based payment.”
- **Governance and safety:** “algorithmic fairness,” “auditability,” “clinical accountability,” “regulatory pathways for AI.”
- **Workflow and experience:** “documentation burden,” “clinician satisfaction,” “user trust,” “AI implementation in primary care.”

Across databases, searches produced large and variable result sets reflecting the rapid expansion of AI-enabled clinical tools documented in recent reviews and policy analyses. From these returns, sources were screened based on conceptual relevance, policy applicability, and alignment with primary care, population health, and integrated behavioral health settings.

### 2.3 Source selection and appraisal

To enhance transparency, sources were considered using the eligibility criteria outlined below and author expertise related to feasibility in real-world primary care and integrated behavioral health settings. Authors applied these criteria, with any differences addressed through discussion.

Selection focused on three core dimensions:

- **Conceptual Relevance** – Direct linkage to AI governance, clinical oversight, risk stratification, or value-based behavioral health integration.
- **Policy Applicability** – Explicit connection to established U.S. reimbursement pathways (e.g., CPT/HCPCS codes, MACRA/MIPS, Medicaid waivers).
- **Workflow Alignment** – Suitability for same-day, high-volume team-based care characteristic of the Primary Care Behavioral Health model.

Because the objective was conceptual integration rather than quantitative aggregation, screening emphasized analytic saturation across domains rather than numerical completeness. Inclusion and exclusion criteria are detailed in Table 1.

**Table 1:** Source selection & appraisal framework.

Criteria	Inclusion logic	Exclusion logic
Evidence type	Peer-reviewed journals (2015–2025); technical reports from authoritative governmental and professional organizations (e.g., CMS, AMA, AHRQ).	Opinion pieces without regulatory or clinical citations; non-indexed or predatory journals.
Methodological rigor	Empirical trials, systematic reviews, or consensus-based policy frameworks with documented methodology and clear data provenance.	Case reports or editorials lacking defined methodology or specific clinical outcomes.
Conceptual relevance	Explicit discussion of AI integration within clinical oversight and value-based care contexts.	Generic AI technical papers or psychology papers without a technology-integration focus.
Policy applicability	Direct linkage to active U.S. billing (CPT/HCPCS) or regulatory pathways (MACRA/MIPS/Medicaid 1115).	International reimbursement models not directly transferable to U.S. billing frameworks.
Workflow alignment	Applicability to same-day, high-volume primary care/IBH models (PCBH/GATHER principles).	Inpatient-only or highly specialized technologies not generalizable to primary care.

### 2.4 Analytic approach

Analysis proceeded in three stages: 1) Mapping AI functions within EHR workflows to identify where AI-generated outputs create discrete, reviewable, and auditable clinical activities requiring clinician oversight; 2) Assessing alignment with reimbursement pathways, including CPT/HCPCS codes, care-management codes, value-based payment arrangements, and Medicaid waiver mechanisms; 3) Evaluating feasibility and safeguards, using criteria related to patient safety, clinical accountability, algorithmic fairness, auditability, workflow integration, and usability in limited resource settings. Insights from these steps were integrated into a systems-based reimbursement framework emphasizing transparency, equity, and operational feasibility.

### 2.5 Evidence gathering, scope, and inclusion criteria

Evidence was selected based on relevance to clinical AI implementation, reimbursement, governance, auditability, and equity in value-based care, with particular emphasis on applicability to integrated behavioral health and primary care settings. Given the absence of a unified evidence base for clinical AI reimbursement, this review integrates literature across health policy, implementation science, health informatics, and equity-focused scholarship.

We included peer-reviewed empirical studies and syntheses, policy analyses, and authoritative grey literature (e.g., CMS, ONC, AMA, and professional societies) that addressed real-world deployment, payment alignment, regulatory considerations, or implementation frameworks relevant to team-based care and value-based payment models. Sources were prioritized based on conceptual relevance, applicability to clinical workflows, and direct policy or implementation implications.

### 2.6 Limitations

Because AI reimbursement and regulation evolve rapidly, evidence is uneven across clinical, policy, and implementation literatures. To strengthen validity, findings were triangulated across peer-reviewed studies, federal and state policy documents, and case examples from health systems implementing AI-enabled tools.

## 3 Background – AI in team care policy context

### 3.1 AI in team care policy context

Despite rapid technical progress, the U.S. healthcare system continues to underperform peer nations on population health, equity, and care coordination (18). This gap has renewed focus on strengthening team-based care and improving the flow of actionable information across settings. AI can support these goals by synthesizing data, aiding detection, and reducing documentation burden, but its impact remains constrained by fragmented reimbursement, variable implementation support, and uneven oversight. Embedding transparency, safety, and auditability into deployment may help AI-generated insights more reliably enhance team-based practice.

Administrative overload remains a major driver of clinician burnout, with 1–2 h of documentation and EHR activity often required for every hour of direct patient care (10). Early evaluations of EHR-integrated automation and ambient AI documentation show 20%–30% reductions in documentation time, with recent clinical trials demonstrating even larger gains (121, 123). Targeting these high-friction workflow tasks can reduce cognitive load and support workforce retention, particularly in primary care and behavioral health teams facing persistent staffing shortages (3, 89).

### 3.2 Defining value-based care – AI in the transition

Value-based care (VBC) developed in response to longstanding concerns that volume-based payment did not consistently improve outcomes, equity, or clinician sustainability (84). By tying reimbursement to measurable quality and outcomes, VBC aims to better align incentives with earlier risk identification, more coordinated workflows, and care that more reliably supports patient health. This orientation reflects the Quadruple Aim, improving patient experience and population health, lowering avoidable costs, and supporting clinician well-being (18). Integrated care models, including PCBH and CoCM, have demonstrated improvements in health and mental health outcomes, and AI alerts may enhance detection and team-based response.

### 3.3 Legislative and policy drivers

Federal policy has accelerated the transition toward value-based care. The Affordable Care Act (ACA) expanded value-based purchasing and established the Center for Medicare & Medicaid Innovation, enabling large-scale testing of alternative payment models and forming the foundation for population-focused delivery reform (5, 18). The Medicare Access and CHIP Reauthorization Act (MACRA) further entrenched this shift by restructuring physician reimbursement into two pathways: the Merit-Based Incentive Payment System (MIPS), which modifies fee-for-service payments based on performance metrics, and Advanced Alternative Payment Models (APMs), including Accountable Care Organizations (ACOs), which assume shared financial and clinical responsibility for defined patient populations (19, 20). By the late 2010s, federal policy momentum made value-based contracts the dominant strategic direction for Medicare and commercial payers alike, with broad consensus that payment would increasingly reward quality, outcomes, and coordinated team-based care (18, 21, 84).

### 3.4 Core infrastructure requirements for VBC transformation

To operationalize AI within value-based care, organizations require reliable data, coordinated workflows, and stable primary care infrastructure, necessitating capacity across three interdependent domains (22). The first is organizational capability, including population health management, strengthened primary care infrastructure, effective care coordination, and analytic functions needed for continuous improvement (3, 20). The second is provider engagement, ensuring that education, incentives, and daily workflows support high-value, team-based practice rather than legacy fee-for-service patterns (16, 24, 81). The third focuses on patient, family, and community partnership, supported by systems that promote whole-person and community health and sustain trusted care teams (1, 23). Academic medical centers face added complexity as they balance specialty care, workforce development, and research missions during this transition.

### 3.5 Team-based care as foundation for AI augmentation

Team-based care remains a core element of high-value primary care, with evidence supporting its role in improving chronic disease management, patient experience, and clinician well-being (3, 24). Because AI tools function as team extenders, supporting detection, documentation, and coordination, they depend on the relational and collaborative structures already present in team-based practice.

### 3.6 Integrated behavioral health (IBH) and the relevance for AI

Integrated Behavioral Health is central to value-based care because behavioral health conditions drive substantial morbidity, mortality, and cost (24, 25). AI may support IBH teams by helping identify behavioral risk, extracting relevant information from unstructured notes, or reducing documentation time, though these applications remain early and require careful oversight. The potential alignment between AI and IBH therefore lies in their shared aim of improving detection, coordination, and follow-up, particularly for high-need patients. However, given the risk of algorithmic bias and variable performance across subpopulations, considerable humility is warranted when interpreting early findings and generalizing beyond well-studied settings (83).

### 3.7 Primary care as the engine of value and a prerequisite for responsible AI

Primary care is increasingly viewed as the organizing hub of value-based care, supporting longitudinal relationships, non-visit-based management, and whole-person care across behavioral, physical, and social domains (18, 26). AI tools depend heavily on these same longitudinal workflows: they require structured data, continuity, and population-level insight to produce reliable, auditable outputs. As scope of practice evolves and primary care teams take on more complex conditions, AI may help summarize information and surface risk, but only when adequate staffing, reimbursement, and workflow stability are in place.

### 3.8 Cultural and educational transformation for VBC and AI adoption

Value-based care does not advance through operational changes alone; it requires a cultural shift in how clinicians learn, collaborate, and interpret data. Similar dynamics appear relevant for AI. Training frameworks that emphasize quality improvement, population health, and team-based practice may prepare clinicians to evaluate AI tools with the skepticism and contextual understanding needed for safe use (2, 27). The theory of situated cognition, learning within real clinical environments, suggests that clinicians benefit from hands-on participation in workflow redesign and technology co-implementation (16). Early work on EHR and AI adoption reinforces this: when clinicians participate in selecting and shaping tools, integration is smoother and unintended consequences are reduced (28, 29).

### 3.9 Challenges and system-level considerations

Despite strong theoretical justification, evidence for VBC's impact is mixed, reflecting variability in implementation, inconsistent incentives, and infrastructure gaps constraining adoption (30). Academic medical centers face additional pressures related to specialty-care demands, research missions, and workforce development, making both VBC and AI implementation especially complex.

### 3.10 Long-term support, sustainability, and preconditions for AI

Sustained value-based care requires long-term investment in leadership, infrastructure, and change management (18, 20). Early experiences with digital tools indicate that AI is no exception. Meaningful integration depends on stable workflows, clear governance, adequate staffing, and reimbursement structures that recognize clinician time spent reviewing and acting on AI outputs. Toolkits and implementation frameworks can offer guidance, but their success appears contingent on engaged leadership and alignment with the principles of high-performing primary care (2, 3). The above highlights the need for continued humility and system-level attention when positioning AI within value-based delivery systems.

### 3.11 Ten domains of high-value primary care: integrating artificial intelligence and integrated behavioral health

The transition from volume-based to value-based care has encouraged primary care systems to explore frameworks that support high-quality, coordinated, and equitable care. The Ten Domains of High-Value Primary Care (1, 3) provide one such structure and offer a helpful lens for examining how artificial intelligence (AI) and integrated behavioral health (IBH) may contribute to ongoing redesign efforts. Although these domains were not originally developed with AI in mind, several aspects appear compatible with emerging digital and team-based innovations (31). When applied together, AI-enabled tools and IBH approaches have the potential to extend team capacity, support coordination, and enhance value-based care delivery (32, 33).

#### 3.11.1 Domain 1: engaged leadership

Leadership engagement is frequently described as an important enabling factor in primary care transformation, particularly in settings balancing clinical, educational, and research demands (2, 16). AI-related work may benefit when leaders frame technology as a support to clinical teams and facilitate co-design processes that consider workflow impact and professional judgment (28). IBH implementation also tends to rely on leadership roles in fostering interdisciplinary trust and shared accountability for mental, physical, and social health outcomes (32, 34).

#### 3.11.2 Domain 2: data-driven improvement

Data-driven improvement remains central to population health management and value-based care (21, 22, 35, 36). AI tools may complement traditional analytics by synthesizing structured and unstructured data to identify risk patterns, behavioral health needs, and gaps in preventive care (14, 29). IBH contributes by documenting behavioral health conditions and social determinants of health (SDOH), thereby enriching the clinical datasets used for risk stratification and care planning (23, 37).

#### 3.11.3 Domain 3: empanelment

Empanelment supports longitudinal relationships and proactive population management (3). AI-supported panel stratification may help teams understand patient complexity, anticipated utilization, and panel size relative to team capacity (38). When IBH is embedded within empaneled teams, behavioral health needs can be addressed in the same relational context, which may help reduce fragmentation (39, 40).

#### 3.11.4 Domain 4: team-based care

Team-based care emphasizes coordinated practice among multiple health professionals (2). AI tools have been characterized as “team extenders,” offering decision support, documentation assistance, and workflow efficiencies that may allow clinicians to focus more of their time on complex or relationship-based work (41, 42). IBH operationalizes team-based care by embedding behavioral health clinicians directly into primary care workflows, contributing to improvements in depression outcomes, adherence, and utilization patterns associated with value-based care (25, 43).

#### 3.11.5 Domain 5: patient–team partnership

Strong partnerships between patients, families, and care teams are considered foundational to value-based care (24). AI-enabled patient tools, such as portals, monitoring systems, and decision aids, may support shared decision-making when designed to enhance clinician–patient communication (31, 41, 94). IBH may strengthen partnership through the normalization of behavioral health treatment within primary care, potentially reducing stigma and increasing accessibility (39).

#### 3.11.6 Domain 6: population health management

Population health efforts aim to monitor and improve outcomes across defined patient groups with attention to quality, cost, and equity (21, 22). AI may be well suited to identifying risk clusters, forecasting utilization, and supporting targeted outreach (14, 38). IBH supports population health by addressing behavioral health conditions, trauma, substance use, and SDOH, factors consistently associated with population-level outcomes (34, 75).

#### 3.11.7 Domain 7: continuity of care

Continuity helps primary care teams provide whole-person, relationship-centered care across the lifespan (3). AI may support continuity by maintaining longitudinal data views, identifying care gaps, and using natural language processing (NLP) to extract relevant details from specialty notes (9, 38). IBH contributes continuity by keeping behavioral and physical health within the same clinical relationship over time (40, 44).

#### 3.11.8 Domain 8: prompt access to care

Access in value-based care includes synchronous and asynchronous modalities, virtual care, and proactive outreach (18). AI tools may support triage, digital messaging, and symptom evaluation, with the potential to increase access for lower-acuity concerns while improving routing for higher-acuity ones (45, 46). IBH enhances access by enabling same-day warm handoffs and reducing delays commonly associated with specialty mental health systems (32, 33).

#### 3.11.9 Domain 9: comprehensiveness and care coordination

Comprehensiveness involves addressing medical, behavioral, and social needs in an integrated manner. AI systems may assist by synthesizing data from EHRs, wearables, patient-reported outcomes, and narrative notes, with NLP helping surface unmet SDOH needs that may not appear in structured fields (14, 80). Predictive algorithms can identify patients at risk for adverse events or disengagement, enabling proactive coordination by nursing, case management, or IBH teams (9, 21, 95). IBH supports comprehensiveness by embedding behavioral health services into routine primary care and contributing to outcomes such as depression remission and adherence.

#### 3.11.10 Domain 10: payment aligned with quality and team-based innovation

Payment alignment is widely recognized as essential for sustaining primary care innovation (1, 17). Many AI functions, such as risk stratification, SDOH extraction, triage support, and documentation assistance, lack clear CPT or HCPCS pathways and are often treated as unreimbursed administrative work (13, 14). Key IBH and PCBH activities face similar challenges, including warm handoffs, team consultation, care coordination, and measurement-based care, all of which have historically been reimbursed inconsistently (11, 33). Existing mechanisms, such as BHI codes, the Collaborative Care Model, and emerging digital medicine pathways, provide partial solutions but vary in scalability across settings and payers (34, 115). Ongoing policy development may help clarify how AI and IBH activities contribute to high-value primary care and how payment models should evolve to support them (26).

Taken together, the policy landscape, care delivery models, and implementation challenges reviewed in this section highlight a central tension: while AI holds significant potential to strengthen team-based, value-based care, its impact remains limited by misalignment across reimbursement, workflow integration, and governance. These gaps are particularly salient in primary care and integrated behavioral health settings, where longitudinal relationships, measurement-based care, and equity considerations are foundational. The following sections build on this context by presenting concrete frameworks that translate these system-level challenges into actionable billing structures, clinical workflows, and policy guidance for responsible AI adoption (Table 2).

**Table 2:** Equity assurance framework: lifecycle stages and safeguards.

AI lifecycle stage	Primary equity risk	Core safeguards	Provider communication role	Feasibility
Micro level Point of care	Automation bias and unequal interpretation of AI outputs by clinicians	• Bias flags in clinical decision support • Uncertainty indicators for AI predictions • Subgroup performance notes embedded in workflow	• Review AI alerts with counter-checks • Document agreement or override decisions • Communicate AI use to patients when relevant	*** High (CDS infrastructure)
Meso level Operational oversight	Disparate access, referrals, or follow-up driven by workflow or resource constraints	• Equity dashboards stratified by patient demographics • Disparity trend alerts for leadership • Workflow audit trails	• Equity champions review dashboard outputs • Communicate findings to frontline teams • Enable workflow adjustments and targeted outreach	** Moderate (value-based programs)
Macro level Governance & models	Model drift and sustained subgroup underperformance over time	• Subgroup validation thresholds • Continuous performance audit logs • Automated retraining or recalibration triggers	• Governance bodies communicate performance findings • Share bias disclosures and mitigation actions • Use standardized reporting (e.g., model cards)	* Lower (requires policy)

This framework identifies equity risks and corresponding safeguards at each implementation level, with provider communication serving as the operational link between technical controls and clinical accountability. Feasibility indicators reflect current infrastructure capacity, highlighting where policy development is needed. Feasibility Legend: *** High (supported by existing CDS governance and safety infrastructure); ** Moderate (commonly supported under value-based quality programs); * Lower (requires clearer regulatory and reimbursement pathways).

## 4 Findings: Framework 1 *Fiscal Framework for Clinical AI in Value-Based Integrated Behavioral Health*

Artificial intelligence (AI) is increasingly integrated into value-based care (VBC) systems and has the potential to support both their implementation and effectiveness. Across primary care, specialty care, and integrated behavioral health (IBH), AI supports risk prediction, documentation, quality measurement, and team coordination (31, 38). Yet these contributions remain structurally invisible in most payment models, because AI is typically treated as information-technology overhead rather than as a clinical contributor (47).

Some pathways are currently feasible within existing CPT, HCPCS, MIPS, APM, and Medicaid waiver structures when AI outputs are clinician-reviewed and auditable. Others represent near-term reforms that would require targeted policy updates, demonstration authority, or payer rulemaking. Where relevant, we explicitly distinguish between mechanisms that are presently operational and those that would require near-term reform, to clarify practical adoption pathways for health systems and policymakers. A coherent billing and policy framework is important to transition AI from back-office software to an auditable, accountable contributing aspect of team care, particularly in IBH and primary care–behavioral health integration (92–94).

### 4.1 The current reimbursement landscape: AI as an unfunded mandate

The number of FDA-cleared AI medical devices now exceeds 1,200, the majority concentrated in radiology (85), where AI has transformed image interpretation and workflow in more than 90% of U.S. health systems (38, 46). Outside imaging and a few EHR-embedded tools, however, adoption remains slow. Health systems consistently cite the lack of direct reimbursement as a key barrier, even when payers acknowledge clinical benefit (47).

Implementation costs extend far beyond software licensing to include digital infrastructure, cybersecurity, workflow redesign, staff training, governance, and ongoing performance monitoring. Large, well-capitalized organizations can absorb these costs; Federally Qualified Health Centers (FQHCs), rural clinics, and Medicaid-focused providers often cannot, exacerbating digital divides and undermining equity goals (18).

Although some AI-enabled services can currently be billed through existing codes, most high-value AI activities, such as risk stratification, SDOH extraction, automated care coordination, and AI-assisted panel management, produce value that is not directly billable.

### 4.2 CPT code evolution: toward billable AI services

The American Medical Association's Digital Medicine Payment Advisory Group (DMPAG), established in 2016, expanded reimbursement pathways for digital health by advancing CPT codes for remote monitoring and AI-enabled services (48). A key milestone was the 2022 introduction of the *Taxonomy of Artificial Intelligence for Medical Services and Procedures*, which classifies AI as assistive, augmentative, or autonomous, ranging from tools that support data processing to those that generate recommendations requiring clinician judgment, and, in limited cases, perform defined tasks autonomously (49). Within existing CPT structures, AI-enabled services are currently feasible for clinician-reviewed documentation and tracking, with Category III codes enabling auditability but not guaranteed reimbursement.

These categories provide shared language for distinguishing machine- from clinician-generated work and may support more transparent, auditable coding structures. AI-related codes now include permanent Category I services, such as coronary CT fractional flow reserve analysis (CPT 75580), and Category III codes for emerging applications like plaque quantification (0623T–0626 T) (50). Although Category III codes do not ensure payment, they enable early documentation of clinical use and may support later transition to Category I status.

Payers have begun exploring reimbursement when three conditions are met:

1. the AI output is stored in an auditable format;
2. a qualified clinician reviews and documents its use; and
3. the AI contribution is attributable for audit.

Within this structure, workflows such as an AI-generated suicide-risk alert followed by a same-day warm handoff, or NLP detection of housing instability triggering referral, can fit within existing coding pathways. Clinicians retain responsibility for decision-making, while AI contributions become visible within reimbursement mechanisms (49, 51).

### 4.3 Medicare and medicaid pathways: aligning AI with value-based reform

Medicare determines coverage for AI-enabled technologies through coding, coverage, and payment decisions governed by medical-necessity standards (117). Although FDA Breakthrough Device designation can accelerate review, Medicare Administrative Contractors ultimately decide whether a device or AI system is reimbursable, and only a subset qualify for New Technology Add-On Payments (52). CMS is attempting to streamline this process through the Transitional Coverage for Emerging Technologies pathway, designed to provide earlier and more transparent coverage for innovations not yet meeting the “reasonable and necessary” threshold (125). The Medicare Access and CHIP Reauthorization Act further embeds technology use in alternative payment models, incentivizing AI systems that improve quality, reduce avoidable utilization, or enhance patient-reported outcomes (53).

State Medicaid programs represent an even more powerful—yet underused—lever for equitable AI adoption. Section 1115 and 1915 waivers allow states to test delivery-system reforms, many now integrating SDOH data, care coordination, and measurement-based behavioral health workflows (97, 122, 124). These waivers have supported major innovations in accountable care organizations, health homes, and coordination programs in FQHCs settings where AI-enabled risk stratification, NLP-based SDOH identification, and closed-loop referral systems may produce population-level benefits (97). AI-supported measurement-based care in underserved regions may allow Medicaid demonstrations to evaluate whether AI reduces preventable utilization, strengthens chronic disease management, and helps narrow equity gaps. Because financing remains a central gatekeeper, aligning Medicaid demonstration authority with AI deployment may accelerate responsible, evidence-generating implementation at scale (47, 54).

### 4.4 Integrating AI into alternative payment models

A sustainable reimbursement approach for AI is unlikely to be achieved through fee-for-service add-on codes alone. Integrating AI reimbursement into existing value-based and population-based payment mechanisms may provide a more durable strategy, reducing the risk of misalignment between how AI tools generate clinical value and how primary care teams are paid for that value. Such alignment may also help mitigate downstream challenges associated with financing models that incentivize episodic activity rather than team-based, longitudinal care. These payment models are currently feasible environments for AI integration, as value is captured at the population or episode level rather than through discrete service codes.

**Accountable Care Organizations (ACOs).** ACOs assume shared financial and clinical responsibility for defined populations. AI tools that improve risk stratification, identify preventive care gaps, or reduce avoidable emergency department use translate directly into shared savings and performance bonuses (30, 38).

**Bundled payments.** Under episode-based bundles, AI-enabled decision support that reduces complications, shortens length of stay, or prevents post-acute readmissions creates measurable value captured within the bundle (30).

**Patient-Centered Medical Homes (PCMHs).** PCMHs emphasize prevention, coordination, and team-based care. AI embedded in EHRs can automate quality reporting, flag high-need patients, support registries, and drive proactive outreach, aligning directly with PCMH recognition standards and incentive payments (15, 20).

**Capitated and population-based payments.** Under per-member, per-month financing, AI that prevents high-cost utilization (e.g., crisis episodes, admissions, readmissions) generates immediate financial return, making capitated systems particularly fertile environments for AI integration (20).

In each model, AI-enabled activities could be explicitly linked to contract metrics so that AI-driven improvements in HEDIS measures, CMS Star Ratings, depression remission, or avoidable utilization are recognized within shared savings and bonus formulas (46, 51).

### 4.5 AI and quality-based payment (MIPS and hospital VBP)

The Merit-Based Incentive Payment System (MIPS) adjusts physician payment based on performance in quality, cost, technology use, and clinical improvement activities. AI tools that demonstrate reliable improvements in process and outcome measures, automate reporting, or support guideline-concordant care may serve as infrastructure for optimizing MIPS scores and avoiding penalties (53, 54).

Hospital value-based purchasing programs, which penalize preventable complications and reward gains in patient safety and patient experience, also create incentives for early-warning and risk-prediction systems such as sepsis detection and readmission modeling. Integrating AI-generated data into measure specifications and automated reporting may allow AI-enabled improvements to be captured more accurately in performance scoring (38).

To support fair attribution, AI systems should produce transparent audit trails documenting when clinical decisions were informed by AI recommendations, whether clinicians accepted or modified those recommendations, and how downstream outcomes differ for AI-augmented vs. non-AI pathways. Such data can strengthen learning health systems while preserving clinician accountability (47).

### 4.6 Integrated behavioral health as a natural home for AI-augmented workflows

Integrated behavioral health (IBH) offers a strong foundation for AI-augmented workflows because it already incorporates the elements, measurement-based care, population health management, stepped-care algorithms, and team-based delivery, that AI most effectively enhances (37, 55). Existing reimbursable infrastructure, including CPT 99492–99494 and HCPCS G2214, requires validated symptom measures, structured psychiatric case review, and registry-based tracking. AI can strengthen these functions through automated screening, real-time registry updates, relapse prediction, and nonresponse detection (31, 56–58, 98).

The Primary Care Behavioral Health (PCBH) model is an important, flexible structure for AI integration. PCBH relies on brief, high-volume behavioral consultations supported by flexible coding pathways, E/M, psychotherapy codes, and BHI 99484, and is organized around the GATHER principles: generalist, accessible, team-based, high productivity, educator, and routinely integrated (18, 33, 39). Each principle maps closely onto AI-augmented workflows: AI tools extend generalist practice by surfacing evidence-based interventions at the point of care; improve access through automated warm-handoff alerts and prioritization queues; strengthen team-based practice via shared dashboards; and support productivity through automated documentation and outcome tracking (46, 56).

Because IBH already depends on registries, systematic measurement, interdisciplinary communication, and accountable workflows, it offers a highly suitable ecosystem for safe, equitable, and auditable AI deployment.

### 4.7 Clinical AI equity framework: from bias risk to equity engine

AI systems are trained on data that sit inside long histories of structural racism, sexism, ableism, and economic exclusion. Without explicit safeguards, clinical AI can silently reproduce patterns such as under-treatment of pain in Black patients, less aggressive cardiac work-ups for women, or lower referral rates for non-English speakers, now at scale and with a veneer of neutrality (59, 99). Yet the same tools can be used to *surface* and reduce inequities when equity is treated as a first-order design constraint rather than a *post-hoc* performance check (100, 101). This section outlines a lifecycle-based equity framework that aligns with existing informatics and policy guidance while remaining practical for value-based care teams (60, 61).

#### 4.7.1 Naming structural harm: how clinical AI can worsen or improve equity

Clinical datasets are not neutral snapshots of “true” health status; they are shaped by who has access to care, which complaints are believed, how pain and distress are coded, and which diagnoses are reimbursable (99). Algorithms trained on such data risk:

- **Encoding historical under-diagnosis and under-treatment** (e.g., lower documented depression rates in men or certain racial groups leading to lower algorithmic risk scores).
- **Reinforcing resource maldistribution**, when models allocate care toward already well-resourced populations because they appear “higher value” in historical claims (60, 102).
- **Normalizing biased documentation and devices**, such as race-adjusted eGFR or pulse oximetry inaccuracies in patients with darker skin (99, 103).

From an equity perspective, the core problem is not simply “model bias” but **system bias being frozen into code**. A distributive justice lens reframes fairness as the *socially just distribution* of health outcomes, risks, and resources across groups, not just parity on narrow performance metrics (60, 88, 101). AI can also help repair inequities when deliberately tasked to: Identify disparities in symptom documentation, referrals, and flag patterns where high-risk patients in marginalized groups are not receiving guideline-concordant care (100). In value-based care, these equity-aware uses align with contractual goals to reduce avoidable utilization and close gaps in preventive and chronic care for groups who have faced marginalization.

#### 4.7.2 Equity by design across the algorithm lifecycle

Advancing equity requires **explicit checkpoints at each phase of the algorithm lifecycle**, from problem formulation through monitoring and re-training (59, 62, 103).

**Problem formulation.** Equity can be be a primary design objective rather than an afterthought. Teams should ask whose problems are being solved, which outcomes matter, and how success will be measured across groups. Inclusion of frontline clinicians, patients, and community partners at this stage reduces blind spots and helps avoid using race or gender as crude proxies for unmeasured structural factors (60).

**Data selection and curation.** Training data should be examined for representativeness, accuracy, and patterns of missingness across racial, ethnic, gender, language, disability, and payer strata (59, 60, 103). Rather than treating biased data solely as “noise” to be corrected, (99) propose viewing them as **informative artifacts** that reveal where systems are systematically failing certain groups. Corrective strategies include collecting new, more representative data; re-weighting under-represented groups; and, where feasible, using federated learning to incorporate data from safety-net systems that have historically been under-sampled in commercial datasets (103).

**Development and validation.** During model building, fairness cannot be bolted on at the end. Developers should conduct stratified analyses of performance across demographic subgroups and examine trade-offs between different fairness definitions, such as equalized odds vs. predictive parity (101, 103). Pre-processing (e.g., reweighting, debiasing labels), in-processing (fairness-constrained optimization), and post-processing methods (e.g., group-wise calibration) all have roles, but their ethical implications should be reviewed with clinicians and affected communities (101).

**Deployment and governance.** At the point of care, the Office of the National Coordinator's HTI-1 rule provides three pragmatic questions that every clinician and health system should be able to answer about deployed AI systems: *What data were used to train this algorithm? How is it intended to be used, updated, and maintained? How does it perform, including on fairness metrics, in our local population?* (60, 61, 80, 88). These questions mirror broader AMIA principles emphasizing transparency, interpretability, safe failure, and continuous model maintenance (61). Embedding this information in model cards and institutional inventories helps demystify AI for clinicians and patients while creating auditable records for regulators and payers.

Monitoring, re-training, and de-implementation continue after go-live. Ongoing attention to performance and equity drift, changes in error rates or disparities as populations and coding practices evolve, is increasingly recognized as an important component of responsible AI oversight (102, 103). When disparities are detected, systematic root cause analysis should ask whether they arise from biased training data, flawed feature selection, context-mismatched deployment, or clinically justified differences. Health systems should define in advance the thresholds that trigger: (a) urgent review, (b) model re-training with updated data, or (c) temporary suspension or full de-implementation (102).

##### 4.7.2.1 Equity assurance with provider-focused communication across the AI lifecycle

Operationally, the equity safeguards described in this framework depend on effective provider-facing communication at each level of AI deployment (81). At the point of care, clinicians must receive bias-related alerts that are timely, interpretable, and actionable, enabling critical review rather than passive reliance. At the operational level, equity dashboards must translate disparity signals into information that clinical teams can understand and act upon, supporting workflow adjustment rather than retrospective reporting alone. At the governance level, clear communication of model performance, limitations, and retraining decisions is necessary to maintain trust and accountability across clinical and administrative stakeholders. Embedding communication requirements within existing quality, safety, and training infrastructure allows equity assurance to function as a practical control layer rather than an abstract principle.

##### 4.7.2.2 Equity trade-offs in under-resourced settings

Rigorous governance, auditability, and clinician-in-the-loop oversight are essential for responsible AI deployment, yet these requirements can create feasibility challenges in under-resourced and safety-net settings. Clinics serving Medicaid-insured, rural, or marginalized populations often face limitations in data completeness, informatics staffing, and audit infrastructure, constraining their ability to implement continuous model monitoring or documentation-intensive workflows. Applying uniform technical and reporting standards across all settings risks unintentionally excluding organizations that may benefit most from AI-augmented care. Equity-oriented implementation therefore requires proportional safeguards: preserving core protections, such as clinician review, transparency, and bias awareness, while allowing flexible documentation pathways, phased audit expectations, or shared resources. Without such accommodation, accountability requirements may inadvertently reinforce existing disparities by concentrating AI adoption within well-resourced systems.

#### 4.7.3 Social infrastructure: teams, training, and community partnership

Technical fixes alone cannot ensure just AI. Social and organizational infrastructure is needed so equity expectations are shared, enforced, and resourced (61, 63).

First, governance bodies should include diverse clinicians, statisticians, ethicists, and community representatives with authority over algorithm approval, monitoring, and de-implementation (59, 61). Aligning these structures with Section 4.8 ensures equity audits, model cards, and bias-response pathways function as prerequisites for reimbursement and continued use.

Second, education and training are high-impact interventions. Most clinicians lack formal preparation in algorithmic fairness, yet must interpret AI outputs for patients. Brief, case-based curricula can teach clinicians to question datasets, validation methods, subgroup performance, uncertainty, and when outputs may be unsafe (41, 100).

Third, human-centered design and community engagement should shape AI development and deployment. Participatory methods and workflow-aligned prototyping help prevent burden and stigma and enable community partners to interpret disparities and co-design mitigation strategies (63, 120).

Finally, in value-based care environments, equity must be tied to payment. Requiring equity impact assessments, stratified reporting, and mitigation plans as conditions of reimbursement creates incentives to invest in this work (60, 102). This shifts AI from a passive risk to an active mechanism for identifying and closing equity gaps (see Table 3).

**Table 3:** Governance, credentialing, & equity requirements for clinical AI in value-based care.

Domain	Core requirements	Purpose in VBC	Key references
1. Safety, reliability, & effectiveness	Rigorous pre-deployment evaluation; assessment of bias, drift, accuracy, and generalizability; alignment with both medical ethics and AI-specific principles (explainability, safe-failure, interpretability).	Ensures AI improves quality while avoiding unintended harm; supports clinician trust and regulatory compliance.	(31, 46, 61, 65, 83)
2. Regulatory coverage gap mitigation	Oversight for non-device AI tools outside FDA authority; local validation, risk controls, and governance committees to manage 21st Century Cures Act exclusions.	Protects against risks in administrative, EHR-embedded, and clinical support tools not covered by medical device regulation.	(47, 64)
3. National clinical AI registry	Documentation of intended use, datasets, validation results, subgroup performance, update history, and deployment context.	Provides transparency, supports reimbursement eligibility, and prevents duplicative pilot testing.	(58, 66)
4. Assurance laboratories (CHAI Model)	Standardized testing environments applying nationally consistent evaluation criteria (e.g., FAVES, fair, appropriate, valid, effective, safe).	Creates trusted national standards for evaluating clinical readiness and equity performance.	(58, 65)
5. Reimbursement-linked credentialing	Annual revalidation; post-deployment monitoring; evidence of real-world effectiveness; registry participation as a prerequisite for payment.	Aligns financial incentives with safe, transparent, lifecycle-sustained AI deployment.	(58, 64)
6. Equity auditing & bias mitigation	Pre-deployment bias audits; stratified performance testing across demographic groups; representative datasets and community involvement.	Prevents AI from perpetuating disparities in access, outcomes, or resource allocation.	(60, 61)
7. Transparent model documentation (Model Cards)	Disclosure of training/validation data, dataset composition, limitations, and algorithmic trade-offs.	Supports interpretability, informed consent, and accountability for downstream use.	(61, 62)
8. Auditability of AI outputs	Timestamped, version-linked, clinician-reviewed outputs, each tied to documented clinical actions and traceable decision trails.	Preserves clinician oversight, enables billing compliance, and supports retrospective evaluation of AI impact.	(46, 47)
9. Local algorithm inventory & monitoring	Internal inventory of deployed models; scheduled performance assessments; detection of drift and unintended effects.	Ensures continuous governance and early correction of harmful trends in AI-augmented care.	(60, 62, 65)
10. Patient & community engagement	Stakeholder participation in data selection, problem definition, and evaluation; transparency in data use and algorithm function.	Builds trust, enhances relevance, and ensures design reflects real community needs.	(59, 60, 64)

### 4.8 Governance, credentialing, and equity requirements

Across all proposed use cases, AI outputs are intended to function only as clinician-reviewed decision support, with auditable documentation, clear attribution, and safeguards designed to mitigate automation bias rather than replace professional judgment. Financing alone cannot ensure responsible AI adoption in healthcare; rigorous governance is an important driver of adoption and impact. Even when reimbursement supports AI deployment, concerns about algorithmic bias, automation bias, data drift, and lack of transparency continue to inhibit clinician and patient trust (31, 47). AI systems used in value-based care can drive safety, reliability, explainability, and fairness throughout their lifecycle. Yet the United States lacks fit-for-purpose regulatory frameworks for many AI tools that fall outside FDA oversight under the 21st Century Cures Act (61, 64). These gaps highlight the need for evaluation protocols grounded both in medical ethics, beneficence, nonmaleficence, autonomy, and justice, and in AI-specific principles such as interpretability, “safe failure,” auditability, and ongoing model maintenance (61, 65).

A national clinical AI registry and coordinated assurance infrastructure represent foundational steps toward trustworthy deployment. Linking reimbursement eligibility to registry participation represents a near-term reform rather than current practice. A registry, analogous to device registries, would document intended use, validation data, subgroup performance, known limitations, and update history for every clinically deployed AI system (64, 66). In parallel, a nationwide network of AI assurance laboratories, as proposed by the Coalition for Health AI, could provide standardized testing environments to evaluate clinical utility, bias, real-world performance, and model drift using consistent FAVES criteria: fair, appropriate, valid, effective, and safe (58). Linking CMS or Medicaid reimbursement eligibility to registry participation, annual revalidation, and post-deployment monitoring would align incentives for developers and healthcare organizations (58). These evaluation mechanisms also support continuous evidence generation through stepped-wedge or interrupted time-series designs to assess real-world impact across populations and care settings (65).

Equity, governance, and auditability requirements help ensure that AI strengthens rather than undermines value-based care. Algorithms need to demonstrate acceptable performance across race, ethnicity, gender, language, disability status, and insurance type, with ongoing monitoring for drift and disparate impact (59). Transparent model documentation, such as algorithm labels or model cards, should outline dataset composition, training methods, limitations, and known trade-offs (61, 62).

For reimbursement, AI outputs generally must be captured in an auditable format, reviewed by a qualified clinician, and linked to a documented care action. These processes create traceable “decision trails” that clarify how clinicians engaged with AI recommendations, including when suggestions were accepted, modified, or overridden (51). Together, these structures aim to support accountable, equitable, and clinician-supervised AI use, reducing the risk of opaque “black-box” decision-making and enabling more reliable integration into value-based care.

This table summarizes ten governance domains—including safety, regulatory coverage gaps, registries, assurance labs, equity audits, reimbursement-linked credentialing, and lifecycle monitoring—each aligned with value-based care priorities.

### 4.9 Reclassifying AI from infrastructure to accountable care team contributions

Taken together, these policy mechanisms create a coherent pathway for integrating AI into value-based care delivery systems. CPT and HCPCS codes begin by recognizing discrete AI contributions as legitimate clinical work. Alternative payment models and VBC contracts then ensure that AI-enabled improvements in quality, equity, and cost are visible in shared savings and performance bonuses. Medicaid demonstration projects extend these gains into safety-net settings by funding AI-augmented IBH and primary care in FQHCs and rural clinics. Credentialing standards and equity audits protect patients and promote transparency, while auditable, clinician-reviewed workflows maintain clear lines of oversight and accountability.

Reframing AI as a reimbursable, auditable component of team-based care aligns financial incentives with the realities of modern clinical practice.

## 5 Findings: Framework 2 *A Workflow Model for Artificial Intelligence–Augmented Primary Care Behavioral Health (PCBH)*

Primary Care Behavioral Health (PCBH) provides a highly conducive environment for integrating artificial intelligence (AI) into frontline care (33). Operating under the GATHER principles, generalist scope, accessibility through same-day care, team-based coordination, high productivity, educator roles, and routine workflow integration, the model depends on rapid detection, efficient triage, and seamless collaboration. AI systems can strengthen these functions when implemented as clinician-governed tools rather than replacements for clinical judgment (29, 52). This section outlines a workflow model for incorporating AI into PCBH based on emerging evidence from primary care, digital health, and behavioral health integration.

Effective implementation is supported by alignment across these domains: workflow and clinical decision pathways, data quality and interoperability, clinician oversight and competency, and reimbursement and auditability mechanisms that support augmented, rather than autonomous, intelligence.

**Figure 2:**
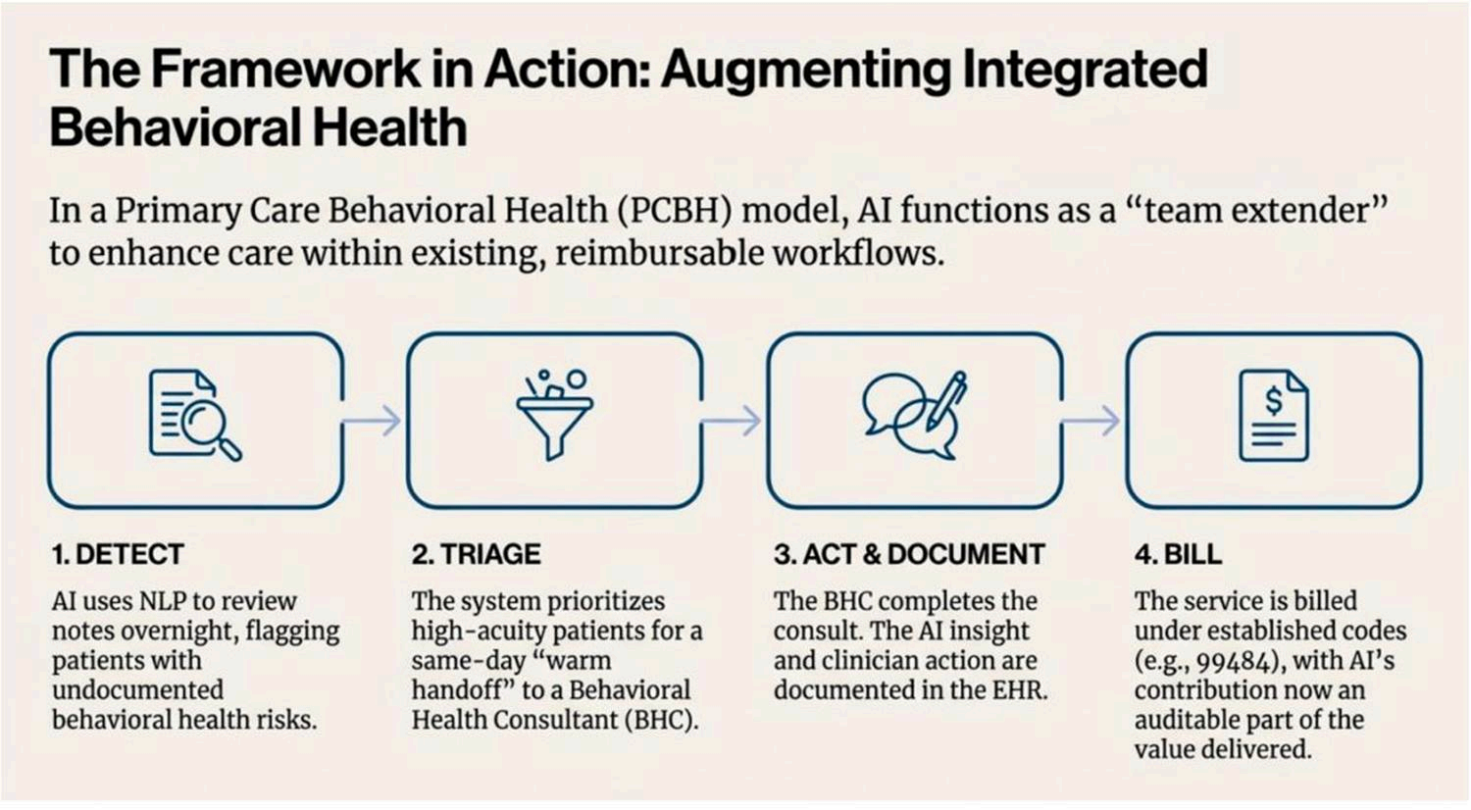
The framework in action: From data inputs to reimbursable AI-augmented care. Is a workflow example of AI-augmented care in an Integrated Behavioral Health (IBH)/Primary Care Behavioral Health (PCBH) model. AI supports overnight detection, triage, and prioritization of high-risk patients. A Behavioral Health Consultant (BHC) conducts the clinical encounter, documents clinician-reviewed AI contributions, and bills existing codes (e.g., 99484). The workflow illustrates how AI integrates into established, reimbursable care pathways.

As illustrated in Figure 2 illustrates a four-step pathway, spanning billing language, clinician review, payment model integration, and governance, that forms the core structure for reimbursing AI-augmented care.

### 5.1 Detection and screening

AI-driven tools can enhance PCBH's generalist function by analyzing longitudinal electronic health record (EHR) data to identify behavioral health, social, and functional risks that may not surface through standard screening. Natural language processing can detect indications of trauma exposure, sleep disturbance, caregiver burden, pain interference, or social determinants such as housing or food insecurity recorded only in free text (80, 94, 99, 104). Automated detection of complex risk patterns can facilitate earlier identification and inform proactive care strategies while preserving clinician authority over final assessment and decision-making (105, 106). Careful monitoring for algorithmic bias can ensure equitable detection across demographic and clinical subgroups (107).

### 5.2 Triage and risk stratification

AI-assisted triage can support PCBH's accessibility mandate by helping prioritize patients based on acuity, engagement likelihood, medical complexity, and social context. Machine-learning models may surface same-day candidates for warm handoffs or identify lower-acuity needs that can be addressed through digital psychoeducation or scheduled follow-up (29). Transparency regarding model logic and limitations allows clinicians to contextualize AI recommendations within professional judgment (108).

### 5.3 Care coordination

PCBH relies on continuous coordination across primary care providers, behavioral health consultants (BHCs), care managers, and community health workers. AI can function as an information management layer, tracking referrals, missed appointments, follow-through, and medication adherence. The management layer can reduce administrative burden and surface gaps requiring team intervention (109). AI-enhanced population registries can also highlight recurring clinical patterns, such as trauma-related presentations or sleep-related distress, which inform huddles, quality improvement priorities, and group-based interventions.

### 5.4 Productivity and documentation

Documentation support is one of the most frequently requested AI applications in primary care, reflecting ongoing documentation burden (104, 109). Automated drafting, structured data extraction, and patient communication templates may reduce clerical work and support PCBH productivity. AI-generated content requires clinician verification and transparent patient disclosure when included in the record (67). These benefits should be balanced against risks of over-automation that may affect clinical skill or interpersonal presence (110).

### 5.5 Clinical education and decision support

PCBH emphasizes the BHC's educator and consultant roles. AI tools can strengthen these functions by providing concise summaries of risk factors, synthesizing chart data, and offering evidence-based intervention suggestions for common behavioral health concerns (94, 106). Importantly, decision support could enhance, and ideally not replace, the therapeutic relationship and shared decision-making is a core aspect of behavioral health care (110). Training clinicians to critically appraise AI outputs is therefore central to safe and effective adoption (68).

### 5.6 Operational integration and governance

Implementation science consistently highlights workflow integration, staff engagement, and documentation standards as preconditions for successful AI deployment (108). Billing workflows would be stronger if they clearly distinguish clinician-reviewed AI insights from automated processes. Governance structures should include ongoing model evaluation, drift detection, and equity audits to monitor real-world performance (107). Multidisciplinary training ensures all team members understand AI's capabilities, limitations, and appropriate uses.

#### 5.6.1 Synthesis

AI-augmented PCBH can strengthen detection, triage, coordination, productivity, and education while preserving clinician judgment and the person-centered ethos of primary care. When embedded into workflows, governed responsibly, and aligned with reimbursement structures, AI has the potential to extend team capacity, support high-value care, and enhance equitable behavioral health integration in resource-constrained settings.

Integrated Behavioral Health models have demonstrated substantial medical cost savings—often $6–$12 for every $1 invested—through reduced hospitalizations, emergency visits, and duplicative testing (111, 112, 118). AI-augmented workflows may reinforce these gains by improving detection, supporting adherence, and reducing missed opportunities for early intervention. Aligned with value-based priorities, these efficiencies may support earlier intervention and reduce avoidable utilization (56, 69).

## 6 Findings. Framework 3 – policy recommendations and implementation roadmap for team-based PCBH care

### 6.1 Defining reimbursable AI services within team-care and PCBH workflows

As AI becomes more embedded in primary care, a central question for team-based and PCBH workflows is when an AI-generated insight should count as reimbursable clinical work. Reimbursement should apply when an AI-generated insight is recorded in the electronic health record, reviewed by a qualified clinician, and linked to a documented clinical action. In PCBH, these instances include AI-assisted detection of behavioral risk from unstructured notes that triggers a same-day consult; natural-language-processing identification of social needs that results in a closed-loop referral; and decision-support tools that inform a brief stepped-care plan attested by the behavioral health consultant. This approach protects professional judgment while recognizing the discrete, auditable contribution of AI to patient care (13, 14).

To avoid the fiction that AI is either “free infrastructure” or a stand-alone billable process, PCBH-aligned policies should explicitly treat clinician-reviewed AI contributions as part of the behavioral health service. The consult note becomes the anchor that ties together the AI signal, the clinician's reasoning, and the resulting action.

**Figure 3:**
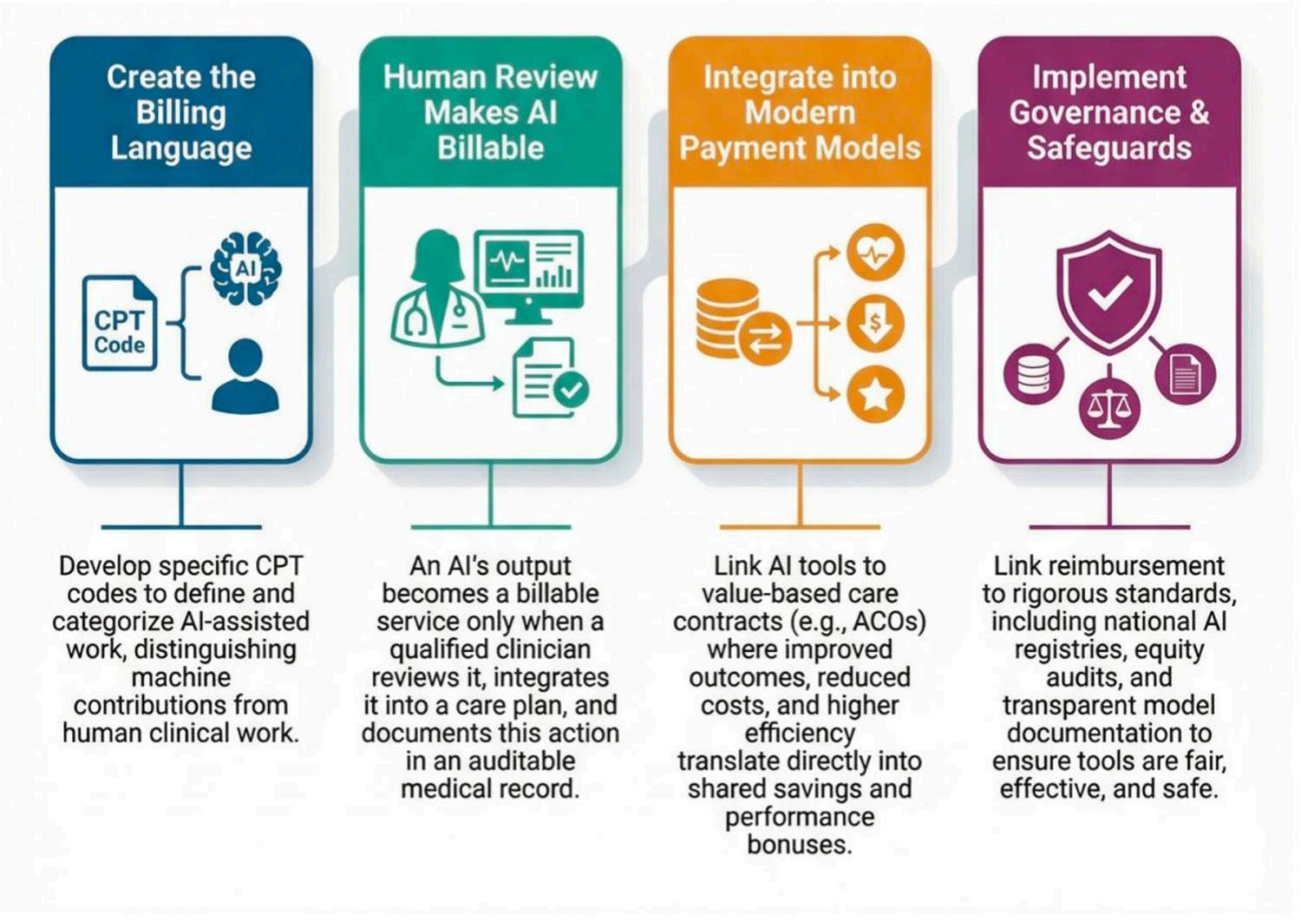
Core conditions required to make clinical AI reimbursable. The four-stage pathway required for reimbursing clinical AI. Reimbursement becomes possible only when (1) CPT and billing language define machine vs. human work; (2) a clinician reviews and integrates AI output into care; (3) AI actions are situated within modern payment models; and (4) governance and safeguards ensure fairness, safety, transparency, and model accountability.

As illustrated in Figure 3 a workflow example of AI-augmented care in an Integrated Behavioral Health (IBH)/Primary Care Behavioral Health (PCBH) model. AI supports overnight detection, triage, and prioritization of high-risk patients. A Behavioral Health Consultant (BHC) conducts the clinical encounter, documents clinician-reviewed AI contributions, and bills existing codes (e.g., 99484). The workflow illustrates how AI integrates into established, reimbursable care pathways.

### 6.2 Embedding AI contributions in value-based contracting

As value-based care expands, behavioral health metrics have become pivotal, and AI is beginning to play an important role in supporting these workflows. Depression outcomes, post-hospital follow-up, avoidable ED use, and SDOH screening increasingly drive contract performance. PCBH-aligned AI workflows can be directly linked to these measures when clinician-reviewed AI outputs lead to documented actions. Aligning AI-generated chart elements with HEDIS and CMS Core Measures allows automated, audit-ready reporting while reducing clerical burden (35, 36, 87). Within this framework, the behavioral health consultant's brief note anchors the chain from AI insight to clinician action to performance attribution in shared-savings arrangement.

Global payment structures and other per-member-per-month models are particularly well suited to AI-augmented PCBH, because investments in proactive risk stratification, registry management, and stepped-care adjustment generate returns that are largely invisible in traditional fee-for-service calculations (70, 113). In practice, measurement-based care (MBC) becomes the operational floor for AI-augmented PCBH: repeated use of validated scales, automated symptom tracking, and AI prompts for stepped-care escalation allow PCBH teams to meet both clinical and reporting expectations with less manual effort (53, 55).

### 6.3 Equity-focused medicaid demonstrations in PCBH settings

PCBH is particularly well suited for Medicaid innovation. Section 1115 and 1915 waivers allow states to fund AI-augmented detection, triage, and team-based coordination in safety-net and rural clinics. Demonstrations can reimburse AI-triaged same-day consults, natural-language-processing-mediated SDOH extraction tied to closed-loop referrals, and registry-based outreach to patients at high risk for disengagement or crisis utilization. To ensure that AI reduces rather than reproduces inequities, waivers should require stratified reporting by race, ethnicity, language, disability, and geography, with predefined thresholds for acceptable performance and mandated remediation plans when gaps appear (14, 71, 72). Medicaid-focused demonstrations can thus function as “learning laboratories” that surface practical implementation models for later diffusion to commercial and Medicare payers.

### 6.4 Credentialing, auditability, and equity safeguards

Reimbursable AI requires formal guardrails. PCBH sites can operationalize these through a combination of registry standards, EHR traceability, and structured equity review.

At the system level, a national or state registry should confirm intended use, validation evidence, and population performance for AI tools approved for reimbursement. Within the EHR, PCBH-related AI outputs should be versioned and timestamped, attributable to model and version, and explicitly linked to the clinician who reviewed the recommendation and the ensuing care action. Mandatory equity audits should track performance across key demographic groups, with remediation plans for performance gaps, particularly in light of documented racial bias in commercial risk algorithms (73).

Many organizations will enact these expectations through multidisciplinary AI oversight committees that include primary care clinicians, behavioral health consultants, patients, data scientists, and equity experts. These bodies review validation evidence, monitor stratified performance, approve model updates, and ensure that AI remains a “co-pilot” aligned with organizational values rather than an opaque black box (29, 45). Together, registry standards, auditability, and local oversight support compliance, legal defensibility, and continuous quality improvement.

### 6.5 Workforce training, supervision, and academic detailing

PCBH's consultation-driven structure aligns naturally with training and supervision around AI use. Curricula should include AI literacy, safe interpretation of decision support, equity auditing, and documentation practices that meet attribution standards. Panel-level dashboards can give residents, fellows, and early-career clinicians feedback on screening reach, symptom trajectories, follow-up intervals, and equity gaps, advancing ACGME competencies in systems-based practice and interprofessional collaboration (27).

Competency-based training can draw on six domains proposed for AI in primary care, foundational AI knowledge, critical appraisal of tools, judgment about when to use them, technical proficiency, clear patient communication, and awareness of unintended consequences, translated into PCBH realities such as same-day consults, curbside consults, and panel-based supervision (68). At the point of care, AI can play an academic-detailing role by surfacing guideline-concordant options and safety advisories in context, while preserving clinician autonomy and making explicit that the behavioral health consultant retains final judgment (12).

### 6.6 Phased implementation roadmap (12–24 months)

A pragmatic timeline allows health systems and payers to move from pilots to scalable practice while maintaining guardrails.

Quarters 1–2 should focus on foundational infrastructure. Health systems and payers specify PCBH-relevant CPT and HCPCS descriptors for AI-assisted services; define EHR audit fields and registry metadata required for reimbursement; and identify two or three early attribution measures such as depression remission, follow-up after hospitalization, or avoidable emergency visits. During this phase, AI tools are mapped onto existing PCBH workflows, with frontline input on alerting, documentation, and division of labor.

Quarters 3–4 shift to pilot deployment. Medicaid and commercial pilots launch in FQHC and rural PCBH clinics, supported by clear coding guidance and performance dashboards. Automated reporting aligned with HEDIS and CMS specifications begins, including stratified equity reporting. AI oversight committees review early performance, clinician feedback, and any emerging safety or equity concerns, adjusting thresholds and workflows accordingly (87).

Year 2 emphasizes scale and dissemination. Pilots expand based on interim findings; stratified performance results are published; participation in AI registries scales; AI-attributed improvements are incorporated into shared-savings formulas; and cross-site playbooks for implementation and workforce training are disseminated. Throughout this period, technical assistance supports smaller, less-resourced PCBH sites through workflow mapping, EHR configuration, billing and coding guidance, and quality-improvement coaching. This support is especially important for practices without in-house informatics capacity (40).

### 6.7 Evaluation framework and guardrails

Evaluation should follow the structure outlined in Section 4 but adapted to PCBH's same-day cadence. Key domains include quality (symptom trajectories, remission, disease control), utilization (emergency department visits, hospitalizations, readmissions), experience (CAHPS and locally developed experience measures), and equity (stratified performance with predefined thresholds and equity-oriented bonus/withhold provisions). Guardrails include transparent model documentation and update logs; clinician-in-the-loop requirements with documented review of AI recommendations; patient information and opt-out provisions where applicable; and rapid remediation plans for bias or safety risks (36, 74). PCBH's continuous-feedback workflow enables timely identification of unintended consequences and rapid course correction (31) (see Table 4).

**Table 4:** Policy framework for reimbursing AI-augmented PCBH care.

Policy lever	What counts/what to pay for	PCBH use case (eligible AI output)	Linked measure or contract domain	Required guardrails	Key citations
CPT/HCPCS: AI-assisted risk assessment	Clinician-reviewed AI risk flags that lead to documented action	AI identifies suicidality → same-day BHC consult completed	Depression response/remission; FUH 7/30	Human-in-the-loop; AI provenance stored in EHR	(13, 23, 36)
CPT/HCPCS: AI-derived SDOH detection	Payment when NLP-identified SDOH leads to a closed-loop referral	NLP flags housing insecurity → referral completed	SDOH screening/closure; avoidable ED use	Equity-stratified reporting; referral audit trail	(14, 35, 36)
CPT/HCPCS: AI-supported care planning	Clinician-attested care plans influenced by AI decision support	AI recommends BA + SSRI titration → plan documented	Depression treat-to-target remission	Model transparency; clinician attestation required	(13, 24)
Shared-savings attribution	Credit for AI-enabled workflows that reduce utilization	Registry-driven outreach lowers ED visits/readmissions	ED utilization; depression response	Independent evaluation; counterfactuals	(8, 36, 79)
Medicaid §1115/§1915 demos	Funding for AI-augmented PCBH workflows in safety-net and rural settings	AI triage+same-day consult; NLP-SDOH+referral loop	FUH; depression remission; SDOH closure	Stratified outcomes; community participation	(23, 71, 72)
Quality alignment (HEDIS/CMS Core)	Reportable AI outputs incorporated into native measure specs	Auto-pack PHQ-9, FUH, SDOH into quality reporting	HEDIS DEP-E; FUH; CMS Core	Conformance testing against measure specs	(35, 36, 87)
National AI registry & credentialing	Payment restricted to validated, listed AI tools	Registered PCBH triage/NLP models with IDs	Eligibility requirement for reimbursement	Post-market monitoring of model performance	(13)
Equity auditing requirement	Mandatory performance stratification+remediation plans	Accuracy by race, ethnicity, language, gender, disability, geography	Equity bonus/withhold; disparity thresholds	Corrective action for biased systems	(14, 73, 83)
EHR auditability & traceability	Payment only when AI outputs are timestamped, versioned, clinician-reviewed	EHR note includes model/version, reviewer, linked action	Claims integrity; legal defensibility	Immutable logs; documented update history	(6)
Training & supervision standards	Recognize supervised AI use in PCBH training and QA workflows	Precepting on AI-informed care plans; use of panel dashboards	CAHPS; fidelity indicators	Clear role definitions; scope-of-practice protections	(12, 27)

### 6.8 Implications for larger, multi-entity systems

Large health systems, academic medical centers, and clinically integrated networks face distinct challenges in adopting AI-enabled workflows. Organizational variability, fragmented governance, heterogeneous EHR platforms, and uneven data quality all limit the ability to scale value-based care (18, 28). These systems require an operating model that treats AI not as a local experiment but as a shared enterprise asset.

A first step is establishing a single “source of truth” for AI-generated outputs, including standardized provenance elements such as model name and version, timestamp, input source, confidence level, and clinician reviewer. Such documentation supports auditability, safety, and billing compliance, echoing the CPT framework emerging for machine-assisted clinical work (49). Standardization also reduces the risk of variable performance across settings, an issue that disproportionately affects minoritized populations and has been documented in algorithmic bias research (14, 73).

Second, payer attribution models can be harmonized across hospitals, affiliated medical groups, and community partners to avoid fragmented incentives.

Finally, multi-entity systems need shared registries for depression, chronic disease, and social determinants of health (SDOH). These registries enable panel-level outreach, proactive stepped-care cycles, and coordinated tasking for primary care and PCBH teams, aligning with national care-coordination guidance (75) and long-standing evidence that clear information sharing improves safety and quality during transitions (76, 77, 119). Robust team-based structures serve as the backbone for these system-level strategies (2).

### 6.9 Coordination within systems: closed-loop workflows

Closed-loop workflows ensure that an AI-generated prompt reliably leads to clinician review, documented action, and measurable outcome. These workflows are foundational to value-based care, which relies on accountability for both process and result (1).

PCBH teams operationalize closed-loop processes through (a) same-day consult pathways triggered by AI risk stratification; (b) concise behavioral health notes that capture AI outputs, clinician actions, and follow-up plans; and (c) registry-driven tasking that ensures reassessment and stepped care (32, 33). Measurement-based care frameworks further reinforce these loops by providing structured, repeatable metrics for decision-making and treatment adjustment (37, 55).

Evidence from the care-coordination literature demonstrates that timely feedback, clear role delineation, and structured transitions significantly reduce avoidable utilization and improve patient safety (23, 76, 77). AI can strengthen these processes by generating structured summaries, pre-populating referral fields, and prompting follow-up actions, while governance structures ensure appropriate oversight (41).

Documentation requirements emerging in digital medicine, particularly machine-assisted CPT standards, also support the operational integrity of closed-loop workflows (49). When implemented responsibly, closed-loop systems reduce clinician burden, mitigate fragmentation, and enhance real-time decision support without compromising autonomy or accountability.

**Illustrative Case Vignette.** During pre-visit screening, an AI tool embedded in the electronic health record (EHR) analyzes PHQ-9 results and recent HbA1c values and flags elevated depressive symptoms alongside suboptimal glycemic control, indicating increased risk for depression–diabetes comorbidity. The primary care provider (PCP) reviews the alert and initiates a same-day warm handoff to integrated behavioral health (IBH), consistent with collaborative care workflows (78, 114).

The IBH clinician assesses moderate depression and medication nonadherence, feeds findings back to the PCP, and collaborates on a treatment plan. AI-generated recommendations—such as patient education materials, group medical visit enrollment, and alerts to pharmacy and nursing—are reviewed and modified by care team members, with clinician documentation preserving accountability and auditability (67).

**Figure 4:**
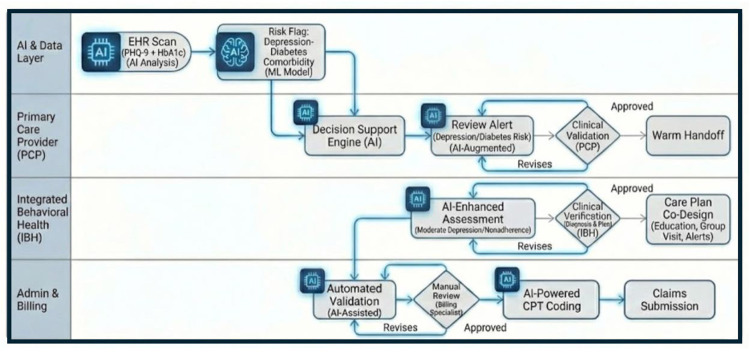
Illustrative AI-augmented care pathway. Is an illustrative AI-supported workflow for a patient with depression–diabetes comorbidity in integrated primary care. The pathway shows how AI-generated screening signals (e.g., PHQ-9 and HbA1c) prompt clinician review, warm handoffs to integrated behavioral health, collaborative care planning, and clinician validation required for auditability and reimbursement under existing Primary Care Behavioral Health (PCBH) and Collaborative Care Model billing structures (CoCM).

As illustrated in Figure 4, AI-generated screening signals (e.g., PHQ-9 and HbA1c) in integrated primary care prompt clinician review, integrated behavioral health handoffs, collaborative care planning, and audit-ready documentation for reimbursement under PCBH and CoCM models.

#### 6.9.1 Coordination across systems: referrals and communication

Referrals across organizations remain a primary point of failure in both fee-for-service and value-based environments. Structured referral summaries, containing the AI-flagged concern, recent measures, relevant SDOH elements, and explicit requested actions, help reduce information loss and improve continuity (75, 76).

Closed-loop referral tracking extends this reliability by converting each referral into a trackable transaction with identifiable states (sent, scheduled, completed, declined), consistent with national quality measures such as timely follow-up after hospitalization (35, 36). AI systems can assist by monitoring referral statuses, surfacing delays, and prompting outreach to patients most at risk for disengagement or poor outcomes.

Supervised agent-to-agent communication allows lightweight AI tools to transmit structured updates across agencies, appointment confirmations, care-plan changes, transportation barriers, while routing all communications back into the EHR for human review. This approach preserves oversight and reduces fragmentation while maintaining a clear audit trail (41).

#### 6.9.2 Streamlining warm handoffs

Warm handoffs represent the cornerstone of the PCBH model, but their reliability is often undermined by unpredictable clinic flow, limited behavioral health capacity, and communication gaps between clinicians. High-performing PCBH programs demonstrate that standardized protocols and role clarity improve the speed and consistency of handoffs, resulting in better outcomes and lower downstream utilization (32, 33, 69).

AI can enhance this process by identifying, in real time, patients who meet criteria for same-day consultation; generating concise, structured briefs that summarize medical, behavioral, and SDOH concerns; and coordinating scheduling availability before the clinician initiates the relational warm handoff. These AI-generated summaries then become part of the behavioral health consult note, supporting stepped-care planning and documentation requirements for VBC (29, 40).

Rather than replacing clinical intuition or interpersonal skill, AI strengthens the reliability and efficiency of warm handoffs by reducing the cognitive and administrative burden on clinicians while preserving the relational core that makes PCBH effective.

### 6.10 Strategic end state

When reimbursement structures, contractual attribution rules, and governance frameworks converge on PCBH-centered workflows, AI transitions from overhead cost to reimbursable teammate. This alignment reflects the principles of high-value care and the foundational logic of the Triple Aim, improving quality, advancing population health, and reducing preventable costs (1, 17).

Modernizing reimbursement pathways enables AI to support population health management, depression care, timely follow-up, and chronic disease outcomes (21, 49, 51). Several possible drivers for this are CPT codes for machine-supported work, attribution adjustments in VBC contracts, and AI-eligible quality incentives. These reforms clarify expectations for AI governance and ensure that benefits accrue across the entire care team.

From an operational perspective, AI reduces delays, strengthens transitions, and supports proactive outreach through registries and structured closed-loop systems, functions that are especially impactful in rural, safety-net, or understaffed environments (38, 46). Team-based care structures remain the organizing backbone for this evolution, providing relational accountability and interdisciplinary problem-solving capacity as AI augments task-based functions (15, 24).

In this strategic end state, the EHR evolves from a retrospective documentation system into an assistive, learning infrastructure. Warm handoffs become more reliable, equity gaps narrow through structured attention to SDOH and bias mitigation, and VBC targets are achieved more consistently across large integrated systems and community clinics alike. AI becomes not an automation tool but an amplifier of relational, team-based primary care, particularly in settings where marginal gains can have transformative impacts.

This table summarizes how CPT/HCPCS codes, value-based contracts, Medicaid demonstrations, quality programs, and governance requirements can be used to reimburse AI-augmented behavioral health services while maintaining clinical oversight and equity safeguards (see Table 5).

**Table 5:** Case vignette: AI-supported screening to reimbursable integrated care.

Role/stage	AI-supported signal or action	Clinician/system response & outcome	Feasibility
Screening (pre-visit)	PHQ-9, HbA1c and comorbidity risk analyzed; registry-level risk flag generated	AI signal logged in EHR; clinician time and validated scales required for CoCM billing (91); AI alone not reimbursable (37, 78)	* Lower
Primary care review	Structured risk summary presented within workflow	PCP reviews alert and initiates same-day warm handoff to IBH clinician; supports collaborative care integration (32, 55)	*** High
Ibh assessment	Depression severity, adherence risk and relapse probability highlighted	IBH clinician validates using structured rating scales; documentation supports CoCM or PCBH billing and measurement-based care (37, 55)	*** High
Care planning & team actions	AI suggests education modules, group visits, pharmacy alerts	PCP leads plan; team actions documented; AI recommendations reviewed and modified as needed; clinician-in-the-loop required (86, 90)	** Moderate
Clinician validation	Role-specific AI summaries generated	Provider documents agreement or override; preserves auditability, transparency and CDS safety (46, 86)	*** High
Billing & transparency	AI surfaces documentation supporting case complexity	Billing verifies CoCM CPT 99492–99494 or PCBH criteria; AI disclosure documented; clinician validation supports compliance and sustainability (56, 78, 82)	*** High

Feasibility Key: *** High=Supported by existing CPT codes and documentation requirements (e.g., Collaborative Care Model CPT 99492–99494). ** Moderate=Feasible but dependent on payer contract, staffing model, or value-based arrangements. * Low/Challenging=Not reimbursable under current CMS rules without clinician-documented work.

## 7 Conclusions

Artificial intelligence (AI) should be recognized as a reimbursable and auditable contributor to value-based care (VBC) when its outputs are embedded within electronic health record (EHR) workflows and governed by explicit safety, transparency, and equity standards. Reframing the EHR as an active clinical partner clarifies how clinician-reviewed AI outputs, risk stratification, natural language processing (NLP) extraction of social determinants of health (SDOH), and stepped-care decision support, constitute discrete, traceable contributions to care rather than invisible digital exhaust. When payers attribute and reimburse these contributions, AI becomes a legitimate contributor to the care team rather than diffuse infrastructure (6, 13).

Centering implementation in Integrated Behavioral Health (IBH), and specifically the Primary Care Behavioral Health (PCBH) model, makes adoption both practical and equitable. PCBH's brief, same-day consultations; population-based scope; and routine, measurement-driven workflows create natural anchor points for documenting AI-enabled actions: triage flags prompting warm handoffs, NLP-identified SDOH generating closed-loop social referrals, and decision support accelerating treat-to-target behavioral health care (24, 32). These workflows map cleanly onto VBC metrics such as depression response/remission, follow-up after mental health hospitalization, and avoidable emergency department use, enabling transparent performance attribution (35, 36, 79).

Deploying AI as a formal contributor to care also requires foundational competencies among clinicians. Effective and safe use of AI requires both conceptual literacy and practical skills. This includes the ability to evaluate model validity, apply outputs appropriately in clinical contexts, communicate transparently with patients, and anticipate unintended consequences (68). These competencies can be paired with multidisciplinary governance structures. These groups bring together clinical, technical, and equity expertise to support performance monitoring, model updates, and alignment with organizational and patient-centered care principles (64).

## 8 Limitations

Three limitations temper these conclusions. First, evidence for AI's real-world population impact varies across settings and algorithms, limiting conclusions about generalizability and long-term sustainability.

Second, equity safeguards increasingly emphasize prospective monitoring rather than retrospective bias checks. However, evidence of their effectiveness remains limited, particularly in domains such as triage and access that are closely tied to existing disparities (14, 73).

Third, documentation and audit requirements can reduce burden when well designed. Yet uneven implementation and limited automation risk shifting administrative work back to clinicians (10).

A further practical limitation is that assigning AI a formal role on the care team is meaningless without trust within the patient–clinician–AI triad. Trust depends on fairness, transparency, and locally validated performance, and is especially critical among historically marginalized groups who have experienced systemic bias and data misuse (41).
